# Not in your usual Top 10: protists that infect plants and algae

**DOI:** 10.1111/mpp.12580

**Published:** 2017-10-11

**Authors:** Arne Schwelm, Julia Badstöber, Simon Bulman, Nicolas Desoignies, Mohammad Etemadi, Richard E. Falloon, Claire M. M. Gachon, Anne Legreve, Julius Lukeš, Ueli Merz, Anna Nenarokova, Martina Strittmatter, Brooke K. Sullivan, Sigrid Neuhauser

**Affiliations:** ^1^ Department of Plant Biology, Uppsala BioCentre, Linnean Centre for Plant Biology Swedish University of Agricultural Sciences Uppsala SE‐75007 Sweden; ^2^ Institute of Microbiology, University of Innsbruck Innsbruck 6020 Austria; ^3^ New Zealand Institute for Plant and Food Research Ltd Lincoln 7608 New Zealand; ^4^ Applied Plant Ecophysiology, Haute Ecole Provinciale de Hainaut‐Condorcet Ath 7800 Belgium; ^5^ The Scottish Association for Marine Science Scottish Marine Institute Oban PA37 1QA UK; ^6^ Université catholique de Louvain, Earth and Life Institute Louvain‐la‐Neuve 1348 Belgium; ^7^ Institute of Parasitology, Biology Centre 37005 České Budějovice (Budweis) Czech Republic; ^8^ Faculty of Sciences University of South Bohemia 37005 České Budějovice (Budweis) Czech Republic; ^9^ Integrated Microbial Biodiversity, Canadian Institute for Advanced Research Toronto Ontario M5G 1Z8 Canada; ^10^ Plant Pathology Institute of Integrative Biology, ETH Zurich, Zurich 8092 Switzerland; ^11^ School of Biosciences University of Melbourne, Parkville, Vic. 3010 Australia; ^12^ School of Biosciences Victorian Marine Science Consortium Queenscliff Vic. 3225 Australia; ^13^ Present address: Station Biologique de Roscoff, CNRS – UPMC, UMR7144 Adaptation and Diversity in the Marine Environment, Place Georges Teissier, CS 90074, 29688 Roscoff Cedex France

**Keywords:** algae, protist, plant pathogens, plasmodiophorids, stramenopiles, phytomonas, phytomyxae

## Abstract

Fungi, nematodes and oomycetes belong to the most prominent eukaryotic plant pathogenic organisms. Unicellular organisms from other eukaryotic lineages, commonly addressed as protists, also infect plants. This review provides an introduction to plant pathogenic protists, including algae infecting oomycetes, and their current state of research.

## Introduction

**Figure 1:**
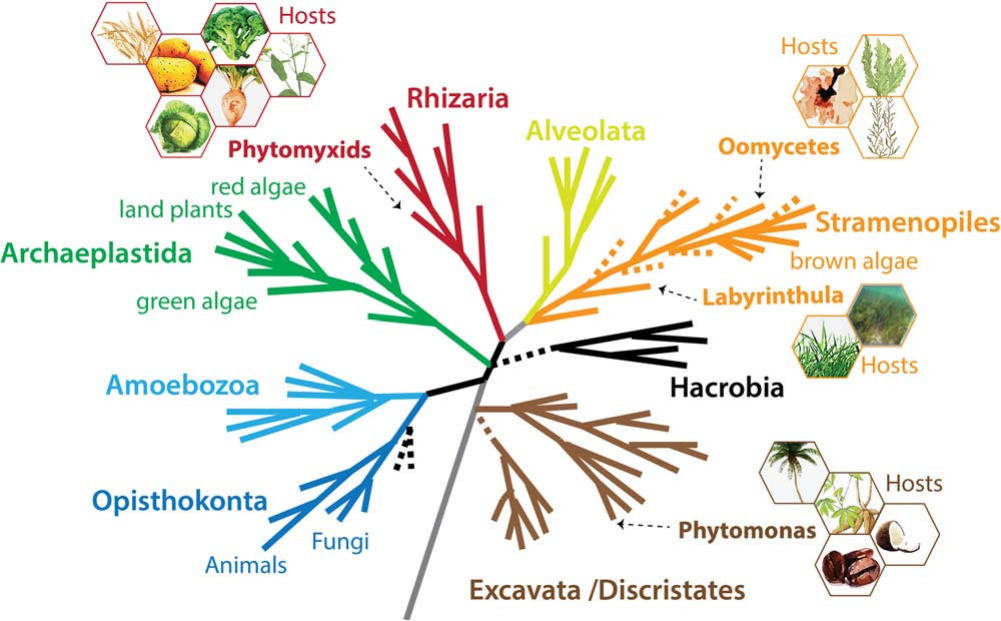
A schematic current eukaryotic tree of life indicating the phylogenetic positions of the eukaryotic plant pathogens outlined in this review. The hexagons show examples of the host species for each pathogen group. The phylogenetic tree was created by S. Baldauf (Uppsala University, Uppsala, Sweden) and reproduced with permission.

*Molecular Plant Pathology* has published a series of the Top 10 most important plant‐pathogenic viruses (Scholthof *et al*., 2011), fungi (Dean *et al*., 2012), bacteria (Mansfield *et al*., 2012), nematodes (Jones *et al*., 2013) and oomycetes (Kamoun *et al*., 2015). The reviews of these major groups of plant pathogens do not cover a selection of protists that infect plants and algae leading to economically important diseases. These ‘non‐standard’ plant pathogens are dispersed across the eukaryotic phylogenetic tree (Fig. 1), often in taxa unfamiliar to many plant pathologists as they are usually not associated with plant infections. In this review, we would like to introduce and raise awareness of such phylogenetically diverse eukaryotic plant pathogens.

We describe diseases caused by these organisms, and the current state of research, especially with respect to their molecular biology and host interactions. We start with *Phytomonas*, plant pathogens in the trypanosomatids in the Excavata supergroup, a group better known as human and animal pathogens. They are followed by Phytomyxea, which are part of the Rhizaria supergroup and include agriculturally important plant pathogens, vectors of phytoviruses and species that infect marine plants and algae (Bulman and Braselton, 2014). Next, *Labyrinthula* are described, plant pathogens in the Stramenopiles, which are phylogenetic basal to oomycetes. Our review also includes marine oomycete parasites of red and brown algae, which impact on the fast growing aquaculture sector (Gachon *et al*., 2010). Advancing research in this field will benefit aquacultural sustainability and our understanding of higher oomycetes because of their basal phylogenetic position inside the oomycetes (Beakes *et al*., 2012).

Whole‐genome or in‐depth transcriptomic data for the species presented here are rare, with the exception of the Phytomyxea and *Phytomonas*. The organisms outlined reflect existing molecular knowledge; nevertheless, we emphasize that there are further important ‘unusual’ pathogens, especially on cultivated algae.

## Excavata – Kinetoplastea Trypanosomatidae – *Phytomonas*

**Figure 2:**
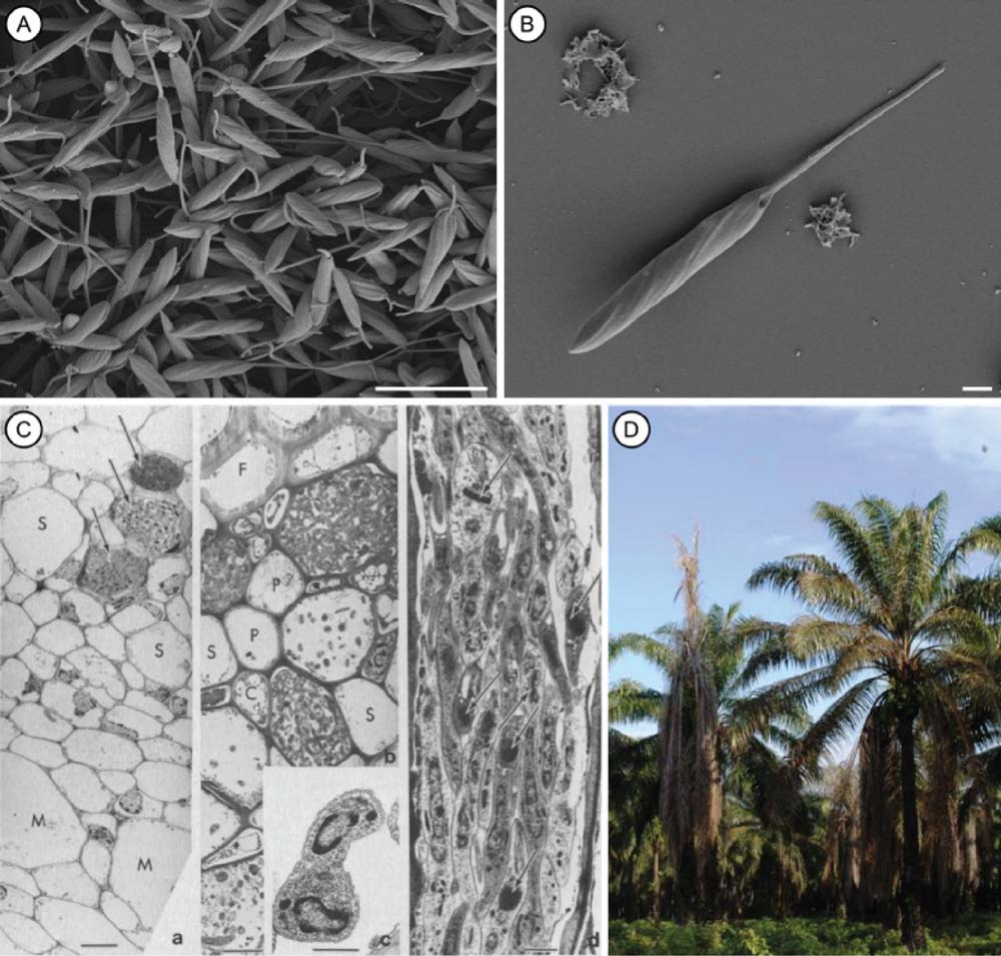
*Phytomonas* sp. and palm infections. (A, B) Scanning electron micrographs of *Phytomonas serpens* cells in culture (scale bars, 10 and 1 μm). (Courtesy of Martina Tesařová.) (C) Transmission electron micrographs of *Phytomonas* sp. flagellates in the phloem of coconut palms affected by hartrot. C, companion cell; F, fibre; M, immature metaxylem; P, phloem parenchyma cell; S, sieve elements free of flagellates. (a) Transverse section of a differentiating vascular bundle, showing recently matured sieve elements filled with flagellates (scale bar, 10 μm). (b) Transverse section of the phloem in palm with advanced symptoms (scale bar, 5 μm). (c) Transverse section of a dividing flagellate (scale bar, 0.5 μm). (d) Longitudinal section of a sieve element filled with flagellates. Arrows indicate the kinetoplast DNA (scale bar, 1 μm). (Reproduced from Parthasarathy *et al*., 1976.) (D) Coconut palms with symptoms of hartrot. (Photograph: Monica L. Elliott, Professor, Plant Pathology, University of Florida, Institute of Food and Agricultural Sciences (UF/IFAS), Gainesville, FL, USA.)

Trypanosomatids are a species‐rich monophyletic group of obligate parasitic flagellates that are usually transmitted by insects. They are best known as agents of human and livestock diseases, such as sleeping sickness, Chagas disease and leishmaniosis, caused by *Trypanosoma brucei*, *T. cruzi* and *Leishmania* spp., respectively (Lukeš *et al*., 2014). Trypanosomatids also include the monophyletic genus *Phytomonas* (Fig. 2), which contains all known plant‐dwelling trypanosomatids, some of which are pathogenic (Seward *et al*., 2016). The ancestral monoxenous lifestyle (development restricted to one host species) of trypanosomatids evolved at least three times independently into a dixenous strategy (Maslov *et al*., 2013) in *Trypanosoma*, *Leishmania* and *Phytomonas* (Lukeš *et al*., 2014). *Phytomonas* spp. are adapted to sap‐sucking insects as primary hosts and plants as secondary hosts (Jaskowska *et al*., 2015). *Phytomonas* spp. were first described from the latex of Mediterranean spurge (*Euphorbia pilulifera*) (Lafont, 1909). Currently, the genus *Phytomonas* includes more than 200 species that colonize over 20 plant families (Camargo, 1999, Jaskowska *et al*., 2015).

*Phytomonas* spp. can be separated into four ecological subgroups based on whether they inhabit the latex ducts, fruits, phloem or flowers of their host plants (Camargo, 1999). Most commonly, *Phytomonas* spp. reside in latex ducts, yet the most pathogenic species are phloem dwelling, such as *P. leptovasorum* and *P. staheli*, which cause coffee phloem necrosis (CPN) and palm wilts, respectively (Jaskowska *et al*., 2015). *Phytomonas leptovasorum* infection triggers multiple divisions of the sieve tubes in coffee roots, leading to CPN. The disease is a potential threat to Brazil as the world's largest coffee exporter, from which CPN has been reported, but never spread (Camargo, 1999). This disease occurs either acutely (plants wither and die within 2 months) or chronically (plants gradually die within a year) (Stahel, 1931).

*Phytomonas staheli* causes wilts of coconut (*Cocos nucifera*) and oil palms (*Elaeis guineensis*) (McGhee and McGhee, 1979). Both deadly wilts, ‘hartrot’ of coconut palms and ‘marchitez sorpresiva’ of oil palms, are characterized by progressive leaf browning, followed by rapid rotting of fruits, spears and roots (Kastelein, 1987; Lopez, 1975). Slow wilt of oil palms (‘marchitez lenta’) manifests as additional chlorosis (Di Lucca *et al*., 2013). Symptomless plants and wild hosts can harbour *Phytomonas* flagellates (Di Lucca *et al*., 2013). Potential disease outbreaks constantly threaten palm cultivation in South and Central America. In one Surinamese district, *Phytomonas* destroyed half of the coconut population (Kastelein, 1987). The latex‐inhabiting *P. françai* is linked to empty roots disease (‘chochamento de raizes’) of the Unha cassava (*Manihot esculenta*) variety, although its pathogenicity remains unclear (Jaskowska *et al*., 2015; Kitajima *et al*., 1986).

The first *Phytomonas* draft genome came from the tomato fruit‐inhabiting *P. serpens* (Kořený *et al*., 2012), which produces no significant systemic disease, but causes yellow spots on fruit (Camargo, 1999). The genomes of the pathogenic phloem‐specific *Phytomonas* strain HART1 from Guyanan coconut and the non‐symptomatic latex‐specific strain EM1 from *Euphorbia* were generated shortly after (Porcel *et al*., 2014). Recently, the genome of the cassava latex‐inhabiting *P. françai* has been announced (Butler *et al*., 2017), which will enable comparative genomics of *Phytomonas* spp. with different host and ecological lifestyles in the future.

The *Phytomonas* genomes are compact, consisting of single‐copy genes, and are almost free of transposable elements and repeats. Therefore they are smaller (≅18 Mb) than most trypanosomatid genomes (26–33 Mb). *Phytomonas* spp. contain only about 6400 protein‐coding genes versus approximately 10 400 found typically in trypanosomatids.

As in other biotrophs, *Phytomonas* metabolism is highly adapted to parasitic lifestyles. These plant pathogens contain fewer genes involved in amino acid synthesis and energy metabolism and fewer protein kinases than the related *Leishmania* and *Trypanosoma* spp. Fatty acids (FAs) are synthesized via elongases instead of *de novo*, as FA synthases are missing (Porcel *et al*., 2014). *Phytomonas* spp. have the unique capacity amongst trypanosomatids to live in the total absence of haem, although they might be able to scavenge it (Kořený *et al*., 2012). In addition, they have lost several cytochrome subunits of respiratory complexes. For energy production, *Phytomonas* may depend solely on glycolysis, whereas other trypanosomatids (at least in part of their life cycle) rely on mitochondrial amino acid metabolism as their main energy source (Jaskowska *et al*., 2015; Porcel *et al*., 2014). As their insect vector(s) feed on carbohydrate‐rich plant juices, *Phytomonas* might not require a switch from carbohydrate to amino acid metabolism. *Phytomonas* spp. contain complete sets of glycolytic enzymes and large numbers of glycosomes, into which glycolysis is compartmentalized (Hannaert *et al*., 2003; Porcel *et al*., 2014). Also unique amongst trypanosomatids, *Phytomonas* spp. possess the capacity to feed on plant polysaccharides using glucoamylase and α‐glucosidase enzymes. In addition, an α,α‐trehalose phosphorylase, acquired by horizontal gene transfer, enables feeding on trehalose, a common sugar in the plant and insect hosts of *Phytomonas* (Porcel *et al*., 2014).

The *Phytomonas* HART1 and EM1 isolates share a majority of genes. However, only the phloem‐restricted pathogenic HART1 encodes invertase genes for the degradation of sucrose (Porcel *et al*., 2014), probably as an adaptation to the abundance of sucrose in the phloem. For both the HART1 and EM1 isolates, 282 secreted proteins were predicted. Their secretomes contain no plant cell wall‐degrading enzymes, which reflects the feeding of the pathogens on extracellular plant fluids. It is unknown whether *Phytomonas* spp. secrete protein effectors, which modulate host plant immune responses. However, several aspartyl proteases that are absent from the genomes of *Leishmania* and *Trypanosoma* are secreted in both *Phytomonas* strains (Porcel *et al*., 2014). These proteases may be involved in *Phytomonas*–host interactions, as seen for oomycete and fungal plant pathogens (Jashni *et al*., 2015). The pathogenic HART1 strain carries five copies of a cathepsin‐like aspartyl protease, derived from duplication events, whereas EM1 has only a single copy. This implies that these enzymes are potential virulence factors (Porcel *et al*., 2014). The gene family of major surface proteases, which are involved in the pathogenicity of *Leishmania*, underwent an expansion in the genus *Phytomonas* (Jackson, 2015). The surface glycoprotein 63 subfamily is present in 20 copies in HART1 and only twice in EM1, a putative adaptation of HART1 to the phloem environment (Jaskowska *et al*., 2015; Porcel *et al*., 2014).

Although the procyclic stage of *Phytomonas* spp. can be easily cultivated, an experimental system including their plant host is not available. Hence, our understanding of how these plant‐dwelling or plant‐parasitizing flagellates interact with their plant hosts is only at an early stage.

Currently, there is no treatment or prevention of the diseases caused by *Phytomonas*, except for the simple extermination of infected plants (Jaskowska *et al*., 2015). However, it has been observed that the tomato (*Solanum lycopersicum*) is relatively resistant to *P. serpens*, as the parasite only causes yellow spots on its fruits, resulting in their lower commercial value. Interestingly, the tomato defensive alkaloids tomatine and tomatidine, surface‐active saponin‐like compounds, induce permeabilization and vacuolization of the parasite (Medina *et al*., 2015). Both alkaloids inhibit the growth of *P. serpens* and therefore represent potential therapeutic agents against these phytopathogens (Medina *et al*., 2015).

## Rhizaria

### Phytomyxea – plasmodiophorids

The obligate biotrophic Plasmodiophorida (plasmodiophorids) belong to the Phytomyxea (phytomyxids) in the eukaryotic supergroup Rhizaria (Fig. 1) (Adl *et al*., 2012; Burki and Keeling, 2014; Burki *et al*., 2010). These organisms infect a wide variety of hosts, including oomycetes and brown algae (Neuhauser *et al*., 2014). Plasmodiophorids cause substantial damage to crops, including brassicas (*Plasmodiophora brassicae*), potatoes (*Spongospora subterranea*) and as vectors of viruses to beets, peanut and monocots (e.g. maize, rice, sugarcane, wheat, sorghum) (*Polymyxa* spp.) and potatoes (*S. subterranea*).

**Figure 3:**
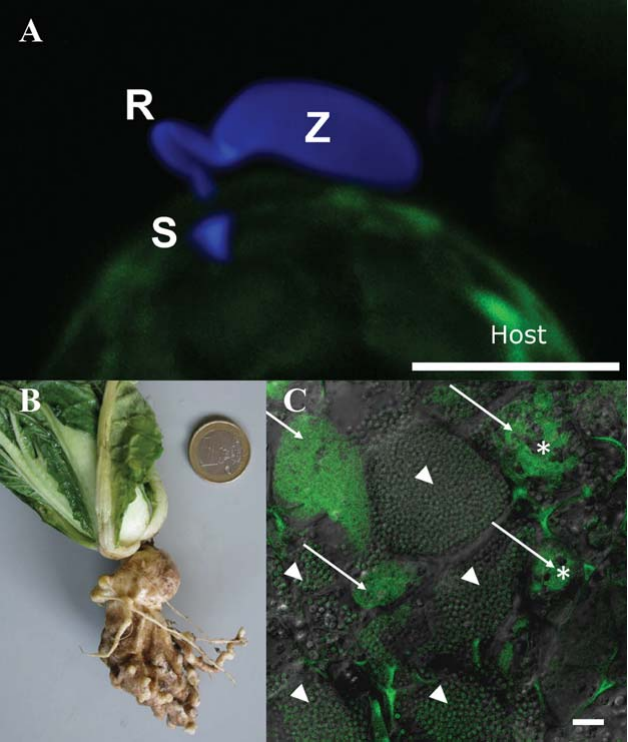
Phytomyxid infection and clubroot. (A) Phytomyxean parasites infect their host via a specialized extrusosome, called a ‘Rohr (R) and Stachel (S)’. The image shows a zoospore (Z) of the phagomyxid *Maullinia ectocarpii* infecting a female gametophyte of *Macrocystis pyrifera* (host). The *M. ectocarpii* spore was stained with calcofluor white and the host is visible via autofluorescence. Bar, 5 µm. (B) Clubroot symptoms on Chinese cabbage. (C) Laser scanning micrograph of *Plasmodiophora brassicae* resting spores (arrowheads) and plasmodia (arrows) in clubroot tissue. Plasmodia of different ages can be distinguished by the presence of typical vacuoles (asterisks), which disappear when the plasmodia start to differentiate into resting spores. Overlay of a light microscopic image and the signal of a *Plasmodiophora*‐specific fluorescence *in situ* hybridization (FISH) probe (green: excitation, 488 nm; emission, 510–550 nm). Bar, 20 µm.

**Figure 4:**
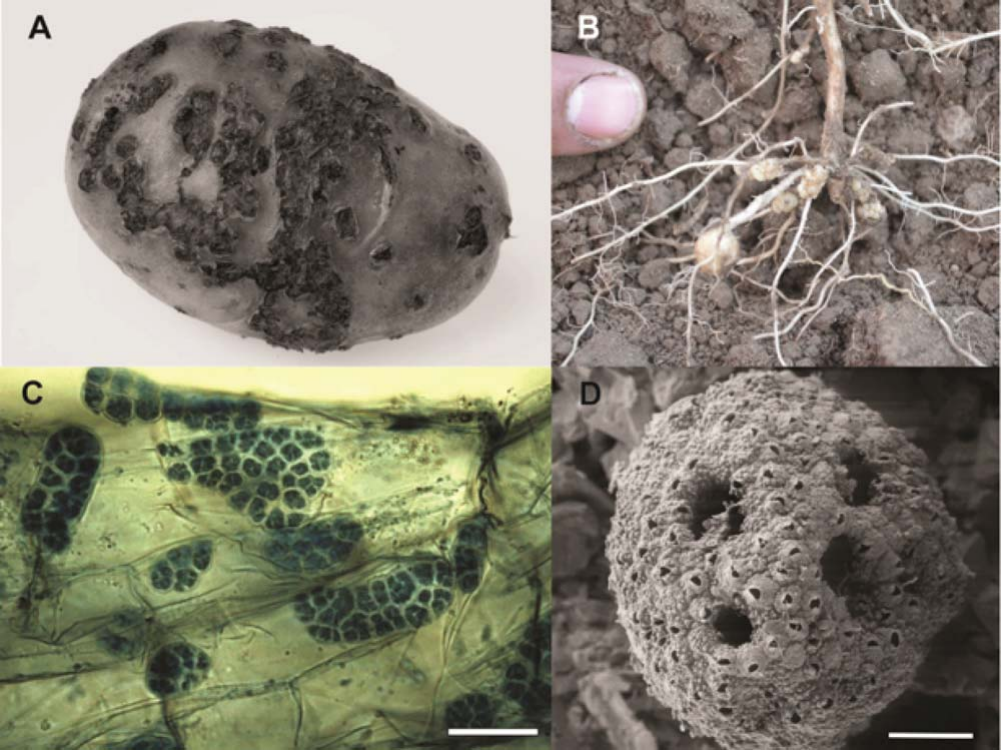
Potato infection by *Spongospora subterranea*. The potato pathogen *Spongospora subterranea* infects host tubers, roots and stolons, resulting in the development of powdery scab lesions (A) and galls (B). These usually appear in potato crops 2–3 months after planting, and mature to release sporosori (conglomerations of resting spores). A sporosorus contains 500–1000 resting spores, each containing a primary zoospore (D; bar, 10 µm). Secondary zoospores formed in zoosporangia (C; bar, 20 µm) emerge through root cell walls, disrupting host nutrient and water uptake.

**Figure 5:**
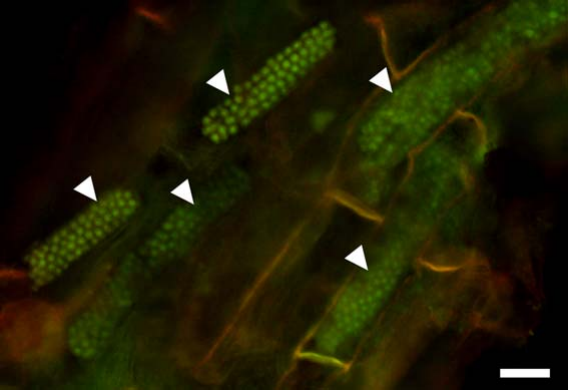
Resting spores of *Polymyxa graminis* in *Poa* sp. Resting spores are arranged in typical, long and cylindrical cytosori (arrowheads). The sample was stained with acridine orange, showing the nuclei of the fully developed resting spores. Epifluorescence micrograph obtained using blue excitation with long‐pass emission (Nikon B‐2A filter) allowing for the detection of DNA. Bar, 20 µm.

The plasmodiophorid life cycle consists of two phases: a sporangial stage leading to short‐lived zoospores, and a sporogenic stage leading to the formation of persistent resting spores (Figs 3, 4, 5). Resting spores give rise to biflagellate primary zoospores which inject their cellular contents into host cells via a ‘Rohr und Stachel’ (Aist and Williams, 1971) (Fig. 3), initiating the sporangial life cycle stage. Multinucleate plasmodia develop and produce (mitotic) secondary zoospores, which can infect host cells and develop sporogenic multinucleate plasmodia that mature into resting spores. In the sporogenic stage, gall‐causing plasmodiophorids induce division and massive enlargement of host cells (for greater detail, see Bulman and Braselton, 2014).

The durability of resting spores and inconsistent chemical control make the management of plasmodiophorid diseases difficult, and biological control efforts are only beginning (Ludwig‐Müller, 2016; O'Brien and Milroy, 2017). Current management mostly relies on the use of resistant host varieties and crop rotation (Bittara *et al*., 2016; Ludwig‐Müller, 2016). Pathogen detection and quantification in soil and *in planta* are important. Sequences of the ribosomal operon [i.e. 18S, 28S and internal transcribed spacer (ITS) ribosomal DNA (rDNA)] are widely used for these purposes (Bulman and Marshall, 1998; Faggian and Strelkov, 2009; van de Graaf *et al*., 2007; Vaianopoulos *et al*., 2007; Ward *et al*., 2005, 2004). Comparison of ITS and rDNA sequences has revealed various degrees of interspecific and intraspecific variation in plasmodiophorid species (Gau *et al*., 2013; van de Graaf *et al*., 2007; Qu and Christ, 2004; Schwelm *et al*., 2016).

#### Plasmodiophora brassicae

*Plasmodiophora brassicae* causes clubroot, a disease that leads to significant losses of *Brassica* oilseed and vegetable crop production worldwide (Dixon, 2009). Rapeseed cultivation for the production of biofuels, vegetable oils, industrial lubricants and rapeseed meal is of great economic importance, with a worldwide production of 27 million tonnes in 2012 (Carré and Pouzet, 2014). Clubroot has long been a major constraint for *Brassica* cultivation. A severe outbreak in 1872 in Russia led to the discovery of *Pl. brassicae* (Woronin, 1877). Clubroot causes crop losses of approximately 10% worldwide, but local losses are often greater (Dixon, 2009). Best practices for control are long crop rotation periods (although resting spores remain infective for decades), liming or cultivation of tolerant *Brassica* crops (Diederichsen *et al*., 2009; Ludwig‐Müller, 2016). Clubroot resistance genes have been identified in *Brassica* genomes (Hatakeyama *et al*., 2013). However, resistance mechanisms are unclear and breakdown of ‘resistance’ has been repeatedly observed (Diederichsen *et al*., 2009; Strelkov *et al*., 2016; Zamani‐Noor, 2017). Breeding for clubroot resistance is complicated as several pathotypes of *Pl. brassicae* exist. Genetic differences exist between *Pl. brassicae* strains, even within individual root galls, and chromosome polymorphism between strains has been suggested (Fähling *et al*., 2003; Graf *et al*., 2004; Klewer *et al*., 2001). However, molecular markers for *Pl. brassicae* pathotypes have yet to be established.

The genome of a European *Pl. brassicae* single‐spore isolate has been generated recently (Schwelm *et al*., 2015), followed shortly after by genomic data for isolates from Canada and China (Bi *et al*., 2016; Rolfe *et al*., 2016). The *Pl. brassicae* genome is small (24.2–25.5 Mb), as a result of a high gene density and few repetitive elements (2%–5%) (Rolfe *et al*., 2016; Schwelm *et al*., 2015). The first single‐nucleotide polymorphism (SNP) cluster analyses of the available *Pl. brassicae* genomes indicated relationships between SNPs, host ranges and regional origins (Rolfe *et al*., 2016). Additional genome sequencing of *Pl. brassicae* isolates should shed light on *Pl. brassicae* genomic diversity and pathotype‐specific features.

The *Pl. brassicae* genomes show similar features to those of other biotrophic plant pathogens. Host dependence is evident, i.e. from a reduced number of biosynthesis genes for thiamine and certain amino acids (Rolfe *et al*., 2016; Schwelm *et al*., 2015). Transporter proteins may aid nutrient acquisition from the hosts (Rolfe *et al*., 2016). The *Pl. brassicae* genome encodes few carbohydrate‐active enzymes (CAZymes). Genes encoding for plant cell wall‐degrading enzymes are also rare, possibly a consequence of the mechanical penetration strategy via a ‘Rohr und Stachel’. However, chitin‐related enzymes are enriched (Rolfe *et al*., 2016; Schwelm *et al*., 2015), which are probably involved in building the chitinous resting spore cell walls (Moxham and Buczacki, 1983).

In root galls, different life cycle stages of *Pl. brassicae* occur simultaneously (Fig. 3), making time course experiments difficult. The transcriptomics of isolated plasmodia show a highly active metabolism, i.e. the high expression of glyoxylate cycle‐related genes suggests a high turnover from carbohydrates and lipids in the plasmodia (Schwelm *et al*. 2015). Lipids start to accumulate in the plasmodial stage and are stored in organelles in the plasmodia and resting spores (Bi *et al*., 2016; Moxham and Buczacki, 1983). The lipids are potential energy sources for resting spores and, as *Pl. brassicae*, like *Phytomonas*, does not contain an FA synthase (Schwelm *et al*., 2015), it might synthesize the lipids from host‐derived precursors.

Depending on the strain sequenced, 553–590 secreted *Pl. brassicae* proteins were predicted. Effector candidates including the amino acid motif RxLR, known from Phytophthora effectors (Kamoun *et al*., 2015), are rare in *Pl. brassicae* (Rolfe *et al*., 2016; Schwelm *et al*., 2015). Crinkler (CRN)‐related proteins were found in *Pl. brassicae* (Zhang *et al*., 2016a), but their functions are unknown. No effector candidates containing the chitin‐binding LysM‐motif, known to interfere with chitin detection in fungal‐plant interactions (Kombrink and Thomma, 2013), were detected in *Pl. brassicae*.

*Plasmodiophora brassicae* infection results in a heavily altered host metabolism (Jubault *et al*., 2013): transcriptional and proteomic changes occur in pathways involved in lipid, flavonoid and plant hormone metabolism, defence responses, and carbohydrate and cell wall synthesis of the *Brassica* hosts (Agarwal *et al*., 2011; Chen *et al*., 2015, Ludwig‐Müller *et al*., 2009; Päsold *et al*., 2010; Siemens *et al*., 2009; Zhang *et al*., 2016b). In *Arabidopsis*, gall formation results from increased host vascular cambium activity combined with significant reduction of xylem development (Malinowski *et al*., 2012). Conversely, higher activity of lignification‐related genes occurs in less susceptible plants (Chen *et al*., 2015; Song *et al*., 2016).

On inoculation, amino acid transport and metabolism vary between tolerant and susceptible hosts, i.e. arginine and proline metabolism are less active in less susceptible *B. rapa* than in susceptible genotypes (Chen *et al*., 2015; Jubault *et al*., 2008; Song *et al*., 2016). Arginine and proline biosynthesis in *Pl. brassicae* also seems to be incomplete (Rolfe *et al*., 2016; Schwelm *et al*., 2015). Similar to other gall‐forming plant diseases, galled roots also provoke hypoxic responses (Gravot *et al*., 2016). Infections by *Pl. brassicae* and morphogenic changes within roots leading to gall formation are accompanied by changes in phytohormone homeostasis of auxin, cytokinin and brassinosteroids (Agarwal *et al*., 2011; Ludwig‐Müller *et al*., 2009; Schuller *et al*., 2014), but the exact mechanisms are not yet known. The contributions of plant hormones in clubroot have been addressed using *Arabidopsis* mutants altered in phytohormone biosynthesis, metabolism and signalling (Ludwig‐Müller *et al*., 2017). In *Arabidopsis*, elevated cytokinins are associated with increased cell division early during infection. When galls are formed, the expression of host cytokinin biosynthetic genes is repressed, as is the expression of host cytokinin oxidases and dehydrogenases (Devos *et al*., 2006; Siemens *et al*., 2006). *Plasmodiophora brassicae*‐produced cytokinins probably play a minor role in cytokinin homeostasis in infected tissues (Malinowski *et al*., 2016). *Arabidopsis* mutants of auxin conjugate synthesis, as well as auxin receptors, were more susceptible to the pathogen (Jahn *et al*., 2013), whereas nitrilase mutants were more tolerant (Grsic‐Rausch *et al*., 2000). A *Pl. brassicae* protein can conjugate auxin and jasmonic acid to amino acids *in vitro* (Schwelm *et al*., 2015), but whether it manipulates host hormones in clubroots is unknown.

Effector‐triggered immunity is likely to be important in host resistance to *Pl. brassicae*. During infection, resistance (*R*) genes and pathogen‐related (*PR*) genes are expressed more strongly in tolerant than in susceptible plants, whereas the pathogen‐associated molecular pattern (PAMP)‐triggered immune response appears to be similar in both host types (Chen *et al*., 2015; Zhang *et al*., 2016b).

One *Pl. brassicae* effector candidate is a predicted secreted methyltransferase, PbBSMT. Biochemical expression assays have shown that this protein can mediate the methylation of salicylic acid (SA) (Ludwig‐Müller *et al*., 2015). PbBSMT might interfere with SA signalling in infected root tissue. SA‐mediated pathways are involved in resistance to *Pl. brassicae* (Agarwal *et al*., 2011; Lemarié *et al*., 2015; Lovelock *et al*., 2013). Accordingly, SA‐responsive gene expression is increased in tolerant hosts (Chen *et al*., 2015; Song *et al*., 2016) and higher SA levels during early infection correlate with resistance (Chen *et al*., 2015; Zhang *et al*., 2016b).

#### Spongospora subterranea

*Spongospora subterranea* causes powdery scab of potato tubers (*Solanum tuberosum*) (Fig. 4A), an important blemish disease in most major potato‐growing regions worldwide. This disease can result in the rejection of whole seed potato lots. The pathogen also causes root galling (Fig. 4B) and is the vector for the *Potato mop top virus* (PMTV, Pomovirus, Virgaviridae) (Merz and Falloon, 2009; Tamada and Kondo, 2013). Root membrane dysfunction, which reduces water uptake and plant growth, has also been attributed to *S. subterranea* (Falloon *et al*., 2016). All of these diseases devalue potato crops, causing potato tuber yield losses of >20% in severely diseased crops (Johnson and Cummings, 2015; Merz and Falloon, 2009; Shah *et al*., 2012). Mature tuber lesions and root galls are filled with clusters of resting spores (sporosori; Fig. 4D), each containing a primary zoospore. Root infection results in the development of zoosporangia (Fig. 4C) producing secondary zoospores. Both types of zoospore infect the host tuber, root epidermis cells and root hairs, and can transmit PMTV.

Disease management is mainly preventative through the use of disease‐free seed tubers and non‐contaminated fields. Powdery scab and root galling susceptibility differ across potato cultivars (Bittara *et al*., 2016; Falloon *et al*., 2003), but no genetic basis of resistance has yet been identified. Metabolites of potato root exudates induce *S. subterranea* resting spore germination, but as l‐glutamine and tyramine have the strongest effects, this might not be host specific (Balendres *et al*., 2016). This may explain reports of primary infection by *S. subterranea* in a range of non‐solanaceous host plants (Merz and Falloon, 2009).

*Spongospora subterranea* ITS rDNA and microsatellite analyses indicate much greater genetic diversity in South American strains (the presumed origin of this pathogen) than elsewhere (Bulman and Marshall, 1998; Gau *et al*., 2013). After the initial dispersal from South America, Europe was probably the main source of spread of *S. subterranea* (Gau *et al*., 2013). Molecular data suggest possible substructures between root gall and tuber scab causing *S. subterranea* lineages from South America (Gau *et al*., 2013). Evidence for recombination in *S. subterranea* is limited, and there is little understanding of sexual recombination in phytomyxids (Bulman and Braselton, 2014).

Limited genomic sequences, including an assembled mitochondrial genome, are available from *S. subterranea* (Bulman *et al*., 2011; Gutiérrez *et al*., 2014, 2016). By comparison, relatively comprehensive *S. subterranea* transcriptomic datasets are available from root galls (Burki *et al*., 2010; Schwelm *et al*., 2015). As for *Pl. brassicae*, the current data suggest intron‐rich genes, a paucity of CAZymes, but an enrichment of chitin‐related enzymes in *S. subterranea*. By contrast, transposable elements are likely to be more common and expressed in *S. subterranea* than in *Pl. brassicae* (Bulman *et al*., 2011; Gutiérrez *et al*., 2014; Schwelm *et al*., 2015). For *S. subterranean*, 613 secreted proteins were predicted – enriched in ankyrin and protein domains – typical of effectors from other plant pathogens. Few are shared with *Pl. brassicae*, but a putative PbBSMT homologue was detected.

Although no genome has been published, genome sequences from *S. subterranea* are being generated. These will identify *S. subterranea*‐specific features and allow research of the transcriptional interaction with its hosts.gg

#### Polymyxa spp

The genus *Polymyxa* includes two morphologically indistinguishable agriculturally important species: *Polymyxa graminis* (Fig. 5) and *Polymyxa betae*. Both differ in their rDNA sequences and host ranges. The host range of *Px. betae* is restricted to Chenopodiaceae and related plants, whereas *Px. graminis* infects mainly graminaceous plants (Legreve *et al*., 2000, 2002). Infection by these obligate root endoparasites is asymptomatic (Desoignies, 2012). Unlike *Pl. brassicae* and *S. subterranea*, *Polymyxa* spp. do not cause root galls on infected hosts, but indirectly cause damage as vectors of plant viruses. *Polymyxa graminis* transmits viruses belonging to *Benyvirus*, *Bymovirus*, *Furovirus* and *Pecluvirus*. They include economically important viruses of different grain crops, such as *Barley yellow mosaic virus* (BaYMV) and *Soil‐borne wheat mosaic virus* (SBWMV), and also cause virus diseases on other cereals, sugar cane and peanuts [*Peanut clump virus* (PCV)] (Dieryck *et al*., 2011; Tamada and Kondo, 2013). *Polymyxa betae* transmits *Beet necrotic yellow vein virus* (BNYVV), causing ‘rhizomania’ in sugar beet (McGrann *et al*., 2009).

*Polymyxa betae* is a well‐defined species, whereas, in *Px. graminis*, five *formae speciales* or six ribotypes exist, with subtype classifications based on ecological, molecular and biological characteristics, including specificity in virus transmission (Cox *et al*., 2014; Dieryck *et al*., 2011; Kanyuka *et al*., 2003; Legreve *et al*., 2002; Smith *et al*., 2013; Vaianopoulos *et al*., 2007; Ward *et al*., 2005; Ziegler *et al*., 2016).

Obtaining genomic data from *Polymyxa* spp. is more difficult than for the gall‐forming plasmodiophorids as high‐density infections with substantial amounts of parasite DNA cannot be identified. *Polymyxa betae* cultures on sugar beet hairy roots (Desoignies and Legreve, 2011) and in its non‐natural host *A. thaliana* (Desoignies and Legreve, 2011; Smith *et al*., 2011) were tested, but were difficult to maintain. A suppression subtractive hybridization experiment identified most currently known *Polymyxa* gene models (Desoignies *et al*., 2014), including 76 *Px. betae* and 120 sugar beet expressed sequence tags (ESTs) putatively involved in the early stages of the host–pathogen interaction. The *Px. betae* ESTs included chitin synthase, polysaccharide deacetylases, ankyrins and galactose lectin domain‐encoding transcripts, proteins which are also enriched in *Pl. brassicae* and *S. subterranea* (Bulman *et al*., 2011; Desoignies *et al*., 2014; Schwelm *et al*., 2015). Genes encoding for profilin and a von Willebrand factor domain‐containing protein were also highly expressed. The sugar beet response to *Px. betae* infection, especially during the plasmodial stage, includes the over‐expression of some defence genes, including those that encode PR proteins or lectins (Desoignies *et al*., 2014).

#### Other Phytomyxea

Other phytomyxids infect freshwater and marine organisms (Neuhauser *et al*., 2011). *Maullinia ectocarpii* (Fig. 3) and *M. brasseltonii* are plasmodiophorids infecting brown algae. *Plasmodiophora diplantherea*, *Pl. bicaudata*, *Pl. halophile* and *Tetramyxa parasitica* cause galls on seagrasses, and, in the case of *T. parasitica*, also other estuarine plants (Bulman and Braselton, 2014; Neuhauser *et al*., 2010). *Spongospora nasturtii* causes crook root on watercress and transmits the *Watercress yellow spot virus* (Walsh *et al*., 1989), impacting watercress cultivation.

### Stramenopiles – *Labyrinthula*

*Labyrinthula* spp. are protists in the Labyrinthulida (Stramenopila), and are phylogenetically basal to oomycetes (Pan *et al*., 2017; Tsui *et al*., 2009). High‐throughput environmental DNA sampling, ITS and ribosomal sequences suggest that *Labyrinthula* spp. are highly diverse, and globally distributed (Bockelmann *et al*., 2013; Collado‐Mercado *et al*., 2010; Martin *et al*., 2016; Pan *et al*., 2017). These organisms are saline tolerant, and can be saprobes, coral inhabitants, endosymbionts of amoebae or endophytic facultative parasites of marine and terrestrial plants (Amon, 1978; Bigelow *et al*., 2005; Pan *et al*., 2017; Sullivan *et al*., 2013).

Marine *Labyrinthula*, such as *L. zosterae*, which causes seagrass wasting disease (SWD) (Sullivan *et al*., 2013), are usually associated with mangrove, macroalgal and seagrass ecosystems (Lindholm *et al*., 2016; Pan *et al*., 2017). Rapid blight disease (RBD) in turfgrasses is caused by the terrestrial species *L. terrestris* in high‐salinity environments, such as salt lakes and golf course turf (Douhan *et al*., 2009; Kerrigan *et al*., 2012). This pathogen may have become important in specialized turfgrass because of increased salinity in irrigation or the use of reclaimed water, causing increased turf salinification (Olsen, 2007; Stowell *et al*., 2005). Both *L. zosterae* and *L. terrestris* vary greatly in virulence to their hosts (Chitrampalam *et al*., 2015; Douhan *et al*., 2009; Martin *et al*., 2016). Although the exact mechanism is uncertain, SWD and RBD manifest through the penetration of host leaf epidermis cells of individual *Labyrinthula* cells.

**Figure 6:**
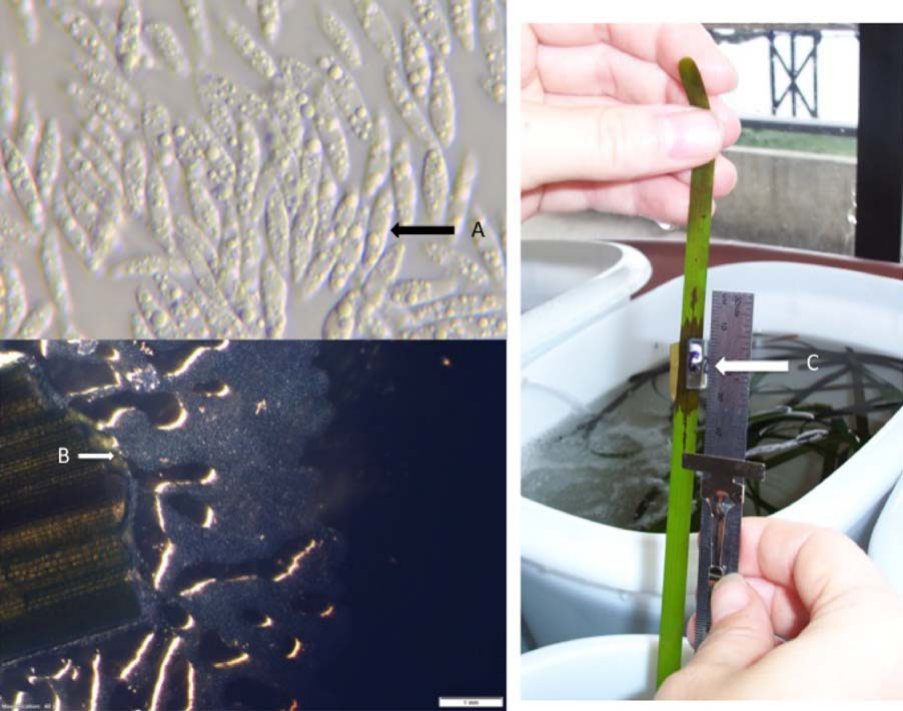
*Labyrinthula* and disease symptoms. (A) Single fusiform cells of the unicellular Labyrinthulomycota *Labyrinthula* protist. (B) *Labyrinthula* cells emerging from a seagrass leaf on serum seawater agar. Cells move through colonies of self‐generated ectoplasmodic networks or ‘slimeways’, a net‐like tube within which *Labyrinthula* are able to move. (C) Symptoms of the seagrass wasting disease 4 days following the artificial infection of seagrass blades.

After infection, *Labyrinthula* spp. destroy the host chloroplasts and advance to neighbouring cells. This creates lesions, sometimes killing entire leaves or plants through interruption of photosynthesis (Fig. 6). These pathogens are therefore found on the edges of progressing infections rather than within the host lesions (Muehlstein, 1992; Olsen, 2007; Sullivan *et al*., 2016). They can be isolated from infected leaf tissues as they emerge from tissues plated onto serum seawater agar solutions (Fig. 6). The individual spindle‐ to oval‐shaped *Labyrinthula* cells move through colonies of self‐generated ectoplasmodic networks or ‘slimeways’, which are thought to originate from specialized organelles called bothrosomes. In conjunction with pseudopodium extension, a net‐like tube is created within which the cells move. The movement of cells occurs through the utilization of an actomyosin system (Preston and King, 2005). The slimeways are also thought to aid nutrient absorption (Vishniac, 1955). *Labyrinthula* cells contain two vacuoles, thought to serve as excretory organs in the cell and may also regulate osmotic pressure, as their presence depends on the environmental salinity (Young, 1943).

The seagrass–*Labyrinthula* pathosystem is the best‐studied relationship for this group. Quantitative polymerase chain reaction (PCR) has shown that *Labyrinthula* spp. occur in most marine eelgrass populations in Europe, but pathogenic species may only cause disease when infection is coupled with host stress (Bockelmann *et al*., 2013; Brakel *et al*., 2014). However, the potential impact of SWD was observed in the 1930s, when *Labyrinthula* killed up to 90% of *Zostera marina*, the most abundant Northern Hemisphere seagrass (reviewed in Muehlstein, 1989; Sullivan *et al*., 2013). Seagrass meadows are ecologically rich and productive marine ecosystems, and important carbon sinks (Christianen *et al*., 2013; Fourqurean *et al*., 2012). They support commercial fish nurseries (Jackson *et al*., 2001) and influence bacterial pathogen populations (Lamb *et al*., 2017). Despite the important ecological and economic roles of their hosts, and widespread evidence of their cause of severe disease, research in *Labyrinthula* pathology is still under development.

*Labyrinthula* spp. tolerate high temperatures up to 28 ºC, but, in tropical and subtropical seagrasses, increased temperature results in reduced virulence (Olsen and Duarte, 2015). Low salinity also inhibits *Labyrinthula* growth (Muehlstein *et al*., 1988), and so seagrass meadows in high‐salinity waters may have an advantage compared with those in truly marine locations (Vergeer *et al*., 1995). The transcriptomic host response to a *Labyrinthula* infection of seagrasses includes the down‐regulation of genes related to reactive oxygen species (ROS) and chitinases, whereas a phenolic acid synthesis gene is highly expressed (Brakel *et al*., 2014). Phenolic metabolites may produce ‘synergistic’ host benefits. Resistance to *Labyrinthula* is density dependent, and diseased leaves have enhanced phenolic metabolite concentrations and these may reduce host susceptibility to *Labyrinthula* (Groner *et al*., 2016; McKone and Tanner, 2009; Trevathan‐Tackett *et al*., 2015). The first seagrass genome (of *Z. marina)* has been published recently (Olsen *et al*., 2016). As a host for *Labyrinthula*, this expands the ability to investigate the genetic and molecular interactions between *Labyrinthula* and seagrass, and to improve our understanding of this potentially devastating pathogen.

### Stramenopiles – oomycetes as algal parasites

Oomycetes cause considerable damage in aquatic crops, including red (Rhodophyta) and brown (Phaeophyceae) algae. Worldwide algal industries have increased dramatically (Loureiro *et al*., 2015). In 2012, global macroalgal production was more than 23 million tonnes (dry weight), with a market value greater than six billion US$ (FAO, 2014). Most of this production (approximately 80%) is used for human consumption, and the remainder for fertilizers, animal feed additives and in medical and biotechnological applications, including biofuel production (Loureiro *et al*., 2015; Stengel and Connan, 2015). Seaweed farming is also often integrated into fish and shellfish aquaculture (Loureiro *et al*., 2015). The total market value for red seaweed reached 3.8 billion US$ (FAO, 2014). Best known in the form of Nori (sushi wrap), *Pyropia* (formerly *Porphyra*) spp. are the most common cultivated red algae. Brown algae are often the predominant primary producers in temperate and cold marine coastal ecosystems (Rodgers and Shears, 2016), and are phylogenetically distant from plants, green and red algae. They differ from red and green algae in cell wall composition (Michel *et al*., 2010), halogen metabolism (La Barre *et al*., 2010), oxylipin synthesis (Ritter *et al*., 2008) and life cycles (Coelho *et al*., 2011). Brown algae include edible seaweeds (e.g. kombu – *Undaria pinnatifida*, wakame – *Saccharina japonica* and sugar kelp – *Saccharina latissima*), and some species are commercially used to produce alginate. Collectively, red and brown algae are affected by many diseases (reviewed in Gachon *et al*., 2010). Because of the economic importance of *Pyropia* cultivation, and the growing economic burden of diseases for this crop (up to 50% of farm costs are spent on disease management: Kim *et al*., 2014), this review focuses on *Pythium porphyrae* and *Olpidiopsis* sp., the two main oomycetes that cause diseases on this crop.

*Olpidiopsis* diseases (previously ‘chytrid rot’) caused Korean Nori farms to lose nearly 25% of their resale value in 2012–2013 (Kim *et al*., 2014), but local losses can be greater (Klochkova *et al*., 2012; Loureiro *et al*., 2015). Environmental factors, such as temperature and seasonality, affect the severity of disease outbreaks.

**Figure 7:**
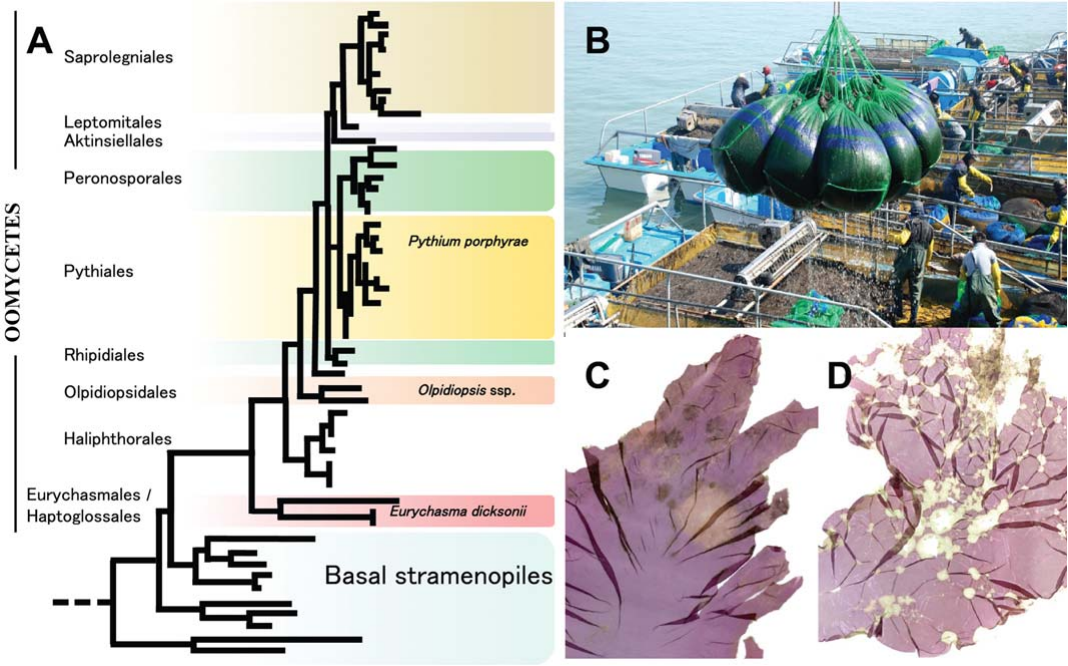
Oomycete phylogeny, *Pyropia* farming, *Pythium porphyrae* and *Olpidiopsis* symptoms. (A) Schematic phylogenetic tree of Oomycetes based on Beakes *et al*. (2012) indicating the positions of the discussed pathogens of marine algae. (B) *Pyropia* seaweed harvest on a commercial farm in South Korea (photograph: H. Kim). (C, D) *Pyropia* blade with lesions caused by *Pythium porphyrae* (C) and *Olpidiopsis* (D) infection. Photographs were originally published in Kim *et al*. (2014) which includes more detailed descriptions of *Pyropia* diseases.

*Pythium porphyrae* causes red rot disease, which is one of the most damaging diseases affecting *Pyropia* farming (Fig. 7) with production losses being greater than 20% (Kawamura *et al*., 2005). Distinct bleached patches on the algal blades characterize the initial infections. The diversity of *Olpidiopsis* is beginning to be described using molecular tools, with the recognition of new species, such as *O. pyropiae* from Korean farms (Klochkova *et al*., 2016; Sekimoto *et al*., 2008), in addition to the Japanese *O. porphyrae*.

#### Olpidiopsis spp

*Olpidiopsis* pathogens are obligate intracellular pathogens with biotrophic lifestyles. During the off‐season of algal cultivation, *Olpidiopsis* may survive in alternative red algal hosts (e.g. *Heterosiphonia* sp.) or as dormant cysts (Klochkova *et al*., 2012, 2016). Germinating zoospores form germ tubes which penetrate algal cell walls. Within the cells, multinucleate walled thalli form, which quickly develop into sporangia, which release zoospores. With advancing infection, host cells break down and lesions in the blades become prominent.

The establishment of *Olpidiopsis* sp. and *Pyropia* pathosystems for research is difficult as the infected host disintegrates in a matter of days. However, with alternative hosts, such as *Heterosiphonia japonica*, stable dual cultures can be achieved (Klochkova *et al*., 2012). *Olpidiopsis* infection in this system is cell type specific, and occurs on the extended rhizoid‐like apical cells. This specificity has been attributed to d(+)‐mannose in host cell walls, indicating a specific lectin–carbohydrate interaction during host–parasite recognition, necessary for zoospore attachment to host cells (Klochkova *et al*., 2012). Until recently, the only available treatment for these diseases was to wash algal blades with acid, a practice now banned because of environmental concerns (Kim *et al*., 2014).

#### Pythium porphyrae

Red rot disease, caused by *Py. porphyrae*, was first described by Arasaki (1947). The disease spreads via zoospores and starts with distinct, small, red patches on the host blades in which the zoospores germinate. The pathogen develops extensive cell‐to‐cell spreading mycelium. Dead host cells change colour to violet–red and green before they degenerate, generating holes that finally destroy entire blades (Fig. 7). Red rot disease management is only effective during the early stages of infection, and PCR methods are important to detect the pathogen early during the algal cultivation period (Park *et al*., 2001, 2006). Disease control involves immersion of cultivation nets into organic acid, freezing of infected cultures and the application of fungicides (Amano *et al*., 1995; Hwang *et al*., 2009; Park *et al*., 2006). These treatments have significant costs and environmental impacts (Park and Hwang, 2015). Disease‐resistant host cultivars are an alternative control strategy. Partially resistant *Pyropia yezsoensis* cultivars, generated from living cells in lesions of infected tissue, have altered cell wall polysaccharide contents (Park and Hwang, 2015). Sulfated galactans (e.g. porphyran) of the algal cell walls may be essential for cyst attachment and infection of *Py. porphyrae*, although the attraction and contact of zoospores are independent of host exudates (Uppalapati and Fujita, 2000). On resistant *Pyropia* sp., cysts with germ tubes frequently grow on the host thallus surfaces without penetration, and show no or delayed induction of appressoria (Uppalapati and Fujita, 2001). Although *Py. porphyrae* zoospores attach and encyst on a number of red algal species, red rot disease only develops on *Pyropia yezoensis* and *Bangia atropurpurea* (Uppalapati and Fujita, 2000).

*Pythium porphyrae* grows best in low‐salinity water, possibly explaining why red rot occurs in farms near river banks (Klochkova *et al*., 2017). The pathogen can also infect and grow on land plants, including Chinese cabbage and rice. *Pythium porphyrae* carried from the land into coastal waters may increase damage in seaweed farms close to river inlets (Klochkova *et al*., 2017). This could enable molecular research on *Py. porphyrae* using the model hosts rice and *A. thaliana*. Genomic data are already available for *Pyropia* hosts (http://dbdata.rutgers.edu/nori/index.php) (Nakamura *et al*., 2013; Wu *et al*., 2014) and are currently being generated for *Py. porphyrae* and *Olpidiopsis* sp.

#### Eurychasma dicksonii

**Figure 8:**
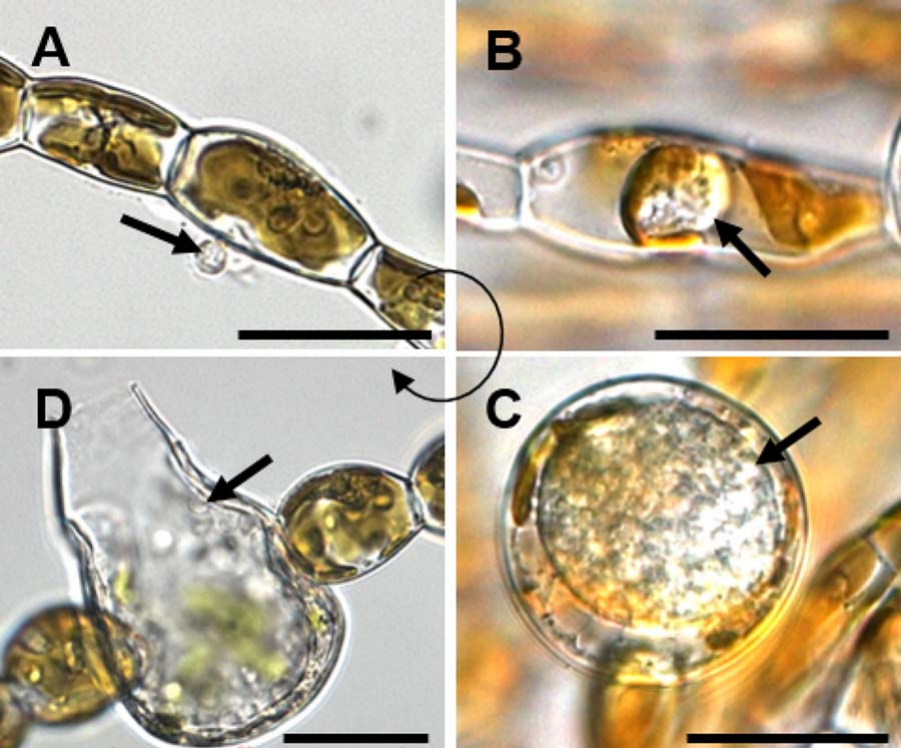
Life cycle of *Eurychasma dicksonii* in its brown algal host *Ectocarpus siliculosus*. (A) A spore (arrow) attaches to the algal surface and injects its content into the host. (B) Within the algal cytoplasm, the *Eu. dicksonii* thallus (arrow) develops which, at the early stage of infection, is unwalled. (C) Later, the pathogen thallus (arrow) has a cell wall and causes hypertrophic expansion of the algal host cell. (D) At the final stage, the complete thallus differentiates into a sporangium from which motile zoospores (arrow) are produced, completing the life cycle of the pathogen. Scale bars equal to 25 μm. (Figure reproduced from Strittmatter *et al*. 2016.)

The most frequently recorded eukaryotic pathogen of brown algae is the biotrophic oomycete *Eurychasma dicksonii*. This phylogenetically basal oomycete (Beakes *et al*., 2012) is geographically widespread, tolerates a broad temperature range (4–23 °C) and infects at least 45 different species of brown algae in laboratory cultures (Müller *et al*., 1999). Similar to *Olpidiopsis* spp., *Eu. dicksonii* is a holocarpic endoparasite (Sekimoto *et al*. 2008). Zoospores attach, encyst and build adhesorium‐like structures at the host surfaces. The parasite cytoplasm is transferred into the host via a needle‐like structure which is associated with the formation of the adhesorium chamber at the host–spore contact point (Tsirigoti *et al*., 2015), similar to the plasmodiophorid ‘Rohr und Stachel’. After penetration, multinucleate non‐walled immature thalli, with double membrane envelopes of host and parasite (Sekimoto *et al*., 2008), develop and expand in the infected host cells, until each cell is almost filled. The plasmodial thallus develops into a sporangium with peripheral primary cysts (Fig. 8), which release biflagellate zoospores through apical exit tubes. The empty cyst walls form a net‐like sporangium, which is a distinctive morphological feature of this pathogen (Petersen, 1905).

*Eurychasma dicksonii* can be cultured in *Ectocarpus siliculosus*, the first brown alga to be genomically sequenced (Cock *et al*., 2010), explaining why the *Eurychasma*–*Ectocarpus* pathosystem is the most thoroughly investigated parasitic interaction in brown algae. A cDNA analysis of *Ec. siliculosus* infected with *Eu. dicksonii* identified 3086 unigenes of oomycete origin. The dataset of *Eu. dicksonii* included 351 proteins predicted to be secreted, but contained no CRN or RxLR effector candidates (Grenville‐Briggs *et al*., 2011). The *Eu. dicksonii* genes included glucanases and a potential alginate lyase, for which no homologues in land plant‐infecting oomycetes have been identified. Alginates and glucans are key components of brown algal cell walls. Similar to higher oomycetes, which secrete cell wall‐degrading enzymes involved in host penetration, this lyase is probably an adaptation to the marine host. In brown algae, β‐1,3‐glucans are usually not part of the cell walls, but are storage polysaccharides. Cell wall modification is a putative host defence mechanism against *Eu. dicksonii*. On infection, cell wall thickening and increased amounts of β‐1,3‐glucans at the penetration site may build physical barriers to pathogen invasion. Large amounts of β‐1,3‐glucan occur at cell surfaces of partially resistant *Ectocarpus* strains (Tsirigoti *et al*., 2015).

Although the infection mechanisms remain largely unexplored, molecular data exist on the host response to infection by *Eu. dicksonii*. Host genes differentially expressed during infection include those encoding for proteins involved in the detoxification of ROS and halogen metabolism (Strittmatter *et al*., 2016). The host genome includes candidate immune receptors of the leucine‐rich and tetratricopeptide repeat families, which quickly evolve via an original exon shuffling mechanism (Zambounis *et al*., 2012). Different hosts display different levels of susceptibility to *Eurychasma* (Gachon *et al*., 2009), and the resistance mechanisms are currently being investigated using cytological and molecular approaches. A targeted movement of host nuclei to pathogen penetration sites has been observed (Grenville‐Briggs *et al*., 2011), and microtubule disorganization in the host occurs only when zoosporogenesis of the pathogen begins (Tsirigoti *et al*., 2015).

## Outlook

Our understanding of eukaryotic plant pathogens is built on studies of fungi, animals (both opisthokonts) and oomycetes (stramenopiles). For the plant pathogens introduced here, the biochemical interactions with their plant hosts are just beginning to be unravelled through the introduction of study systems (e.g. the *Eu. dicksonii*–brown algae interaction) or the generation of reference genomes (*Pl. brassicae*, *Phytomonas* spp.). This will allow the presented pathogens to take a more prominent place in the molecular plant pathology field in the coming years, create deeper insights into how these pathogens interact with their hosts and how they have evolved. This should finally lead to new strategies for the control of these pathogens.

## Author Contributions

A.S. and S.N. initiated and organized the manuscript, and the other authors are listed alphabetically. Section contributions are as follows: *Phytomonas* (J.L., A.N., A.S.), plasmodiophorids (A.S., S.N., S.B., R.E.F., U.M., N.D., A.L.), *Labyrinthula* (S.N., B.K.S.), oomycetes (C.M.M.G., M.S., A.S., S.N., J.B., M.E.). All the authors read the manuscript and agreed to publication.
